# On Denoising Diffusion Probabilistic Models for Synthetic Aperture Radar Despeckling

**DOI:** 10.3390/s25072149

**Published:** 2025-03-28

**Authors:** Alec Paul, Andreas Savakis

**Affiliations:** Department of Computer Engineering, Rochester Institute of Technology, Rochester, NY 14623, USA; asp6244@rit.edu

**Keywords:** denoising diffusion probabilistic models, image denoising, SAR image despeckling

## Abstract

Synthetic Aperture Radar (SAR) images are significantly degraded by multiplicative speckle noise, making their analysis and interpretation challenging. Recently, Denoising Diffusion Probabilistic Models (DDPMs) have demonstrated success in image generation and image enhancement tasks, such as denoising and super-resolution. This paper examines the performance of DDPMs for SAR despeckling using both synthetically speckled and real SAR images. Proposed modifications to the DDPM framework include (i) using a non-uniform step size and spread, along with early stopping in the denoising process, and (ii) sample aggregation by training of a secondary aggregating U-Net to extract additional performance from the partially denoised DDPM samples. Both of the proposed modifications improve accuracy and reduce inference time by utilizing fewer iterations. Various datasets, training methodologies and evaluation metrics are utilized to comprehensively assess the effectiveness of DDPM models for SAR despeckling and benchmark their performance against state-of-the-art SAR despeckling techniques, focusing on accuracy, training time, and evaluation time. Our findings provide insights into the benefits and limitations of DDPMs in the context of SAR despeckling. While diffusion models for SAR produce sharper and more realistic imagery, they sometimes hallucinate and result in lower quantitative performance compared standard U-Net denoising, highlighting the need for better metrics and improved techniques.

## 1. Introduction

Synthetic Aperture Radar (SAR) imagery consists of radio-frequency images of the earth’s surface taken from satellites or high-altitude aircraft. These images are created by sending a radio signal toward the ground and measuring how much of the signal is reflected back. SAR has advantages over optical imagery in situations that require imaging through clouds or at night, making it valuable for military operations and disaster-relief efforts. However, because SAR uses long wavelengths, its signal can penetrate translucent objects like leaves. This causes the signal to reflect and scatter multiple times before returning to the radar sensor. This scattering effect, known as “backscattering”, produces a type of noise called speckle, which appears as a grainy “salt and pepper” pattern in the image [1].

Speckle noise is inherent to all SAR imagery. Since the advent of SAR imagery, post-processing and machine learning algorithms are considered to remove speckle. Early efforts to despeckle SAR images consisted of spatial domain filters that often oversmoothed the image. More advanced approaches have evolved using convolutional neural networks (CNNs) and generative adversarial networks (GANs).

Most recently, diffusion models have become the state of the art for image generation and super-resolution. Jonathan et al. [2] introduced diffusion as Denoising Diffusion Probabilistic Models (DDPM), and Dhariwal and Nichol [3] showed that it can outperform GANs in image synthesis tasks. Diffusion has since made advances in the fields of image super-resolution, in-painting, and restoration. Additionally, Perera et al. [4] have shown it to be useful in SAR despeckling, but a fine-tuning of the diffusion architecture specifically for SAR despeckling has yet to be studied. Thus, this project has three primary objectives:

- Propose a DDPM method that stops the reverse diffusion process early and averages the results of the last few iterations for improved performance.
- Propose a novel DDPM method that trains a secondary aggregating U-Net that produces a despeckled image from the partially denoised results of all DDPM iterations.
- Evaluate the performance of DDPM models for SAR despeckling using both synthetically speckled and real SAR images.
- Identify the benefits (sharper, more realistic images) and limitations (hallucinating incorrect structures) of DDPM models for SAR despeckling.

## 2. Related Work

Speckle is a component of interference noise resulting from backscattering effects of multiple SAR targets. It is present in all representations of SAR data, including amplitude data, phase data, and backscattering coefficients that represent the true reflectivity of the surface. Each representation includes useful information regarding the captured scene.

However, because all SAR images contain some amount of speckle noise, clean SAR images are difficult to acquire for training datasets. Today’s methods typically consist of one of two approaches: multitemporal or synthetic images. Multitemporal SAR images are produced by averaging several passes over the same region of land. However, data are often corrupted by changes in the image between passes, such as vehicle movement and seasonal variations in vegetation. Such problems make it difficult to collect truly clean multitemporal images.

Alternatively, adding synthetic speckle noise to clean optical images is used to obtain datasets for training. Optical aerial image datasets are abundant and very clean, which means that these images can be speckled to produce a dataset of pseudo-SAR images using a model of speckle noise.

Models trained on synthetically speckled optical images do not utilize any phase information since none is available. Therefore, during testing of real SAR images, phase data are omitted and either amplitude data or the backscattering coefficients are used.

Generally, speckle noise is multiplicative and can be expressed as

(1) Y=X·N

where *X* is the clean image, *Y* is the speckled image, and *N* is the speckle noise component. The speckle noise *N* is commonly known to follow a Gamma distribution [5] with a probability density function defined as

(2) $$p\left(N\right)=\frac{L^{L}N^{L-1}e^{-LN}}{\Gamma (L)},L\leq 1,N\geq 0$$

where *L* is the number of looks for multilook images, and Γ is the Gamma function with a unit mean and variance of 1/L.

The earliest efforts to despeckle SAR images primarily involved spatial domain filters such as the Lee filter [6], Kuan filter [7], and Gamma maximum a posteriori (MAP) filter [8]. These basic methods typically use a sliding window that despeckles the center pixel by calculating a correlation or weight from all pixels within the window. However, they often result in oversmoothed edges and loss of important features. While these methods are fast, their despeckling performance deteriorates when applied to larger window sizes.

Over the past two decades, classical despeckling algorithms have seen significant advancements. In 2005, Buades et al. [9] introduced the Non-Local Means (NLM) filter. Unlike traditional methods that rely solely on local information (e.g., neighboring pixels) to estimate true pixel values, NLM leverages the redundancy across the entire image by analyzing patches. Debov et al. [10] expanded on this block-based approach with BM3D, incorporating collaborative filtering in a three-dimensional (3D) transform domain. BM3D is particularly effective at removing Gaussian noise while preserving fine details and textures. Deledalle et al. [11] further developed patch-based methods with their “Probabilistic Patch-Based” (PPB) filtering, introducing a probabilistic framework to enhance robustness and accuracy in noise reduction. In 2012, Parrilli et al. [12] adapted BM3D specifically for despeckling SAR images, an approach that remains widely used today. Cozzolino et al. [13] improved the inference time of this patch-based approach by probabilistic search termination and look-up tables.

Ever since machine learning became computationally efficient enough for mainstream use, significant research has focused on its application in SAR despeckling. Today, many state-of-the-art methods rely on convolutional neural networks (CNNs) for this task. SAR-CNN [14] was the first to leverage CNNs for SAR despeckling, training on multitemporal SAR images to estimate speckle noise as the residual rather than directly reconstructing the clean image.

In 2015, Ronneberger et al. introduced U-Net [15], a CNN architecture designed for image segmentation, particularly in medical imaging. Its “U”-shaped structure includes an encoder for context capture via downsampling and a decoder that restores spatial resolution with upsampling, using skip connections to retain spatial details. Lattari et al. [16] adapted U-Net for SAR despeckling, pretraining it on synthetically speckled images from the UC Merced Land Use dataset [17] and fine-tuning on multitemporal Sentinel-1 SAR images [18], using a total variation (TV) loss function. Vitale et al. developed MONet [19], a custom CNN architecture for SAR despeckling with a multi-objective loss function. Their loss combines the mean squared error (MSE) of the image, MSE of the image gradient, and Kullback–Leibler divergence, aiming to balance spatial detail preservation, speckle statistical properties, and strong scatterer identification. They trained their model on synthetically speckled images from the UC Merced Land Use dataset [17].

In 2022, Liu et al. introduced MRDDANet [20], an advanced CNN architecture that uses parallel CNNs with varying kernel sizes for multiscale feature analysis, incorporating both pixel-wise and channel-wise attention mechanisms. Their model was also trained on the UC Merced Land Use dataset [17] with synthetic speckle, but utilizes an $L_{1}$-norm loss function. Shortly after [20], Ko and Lee proposed SAR-CAM [21], a multiscale CNN autoencoder architecture. To address the local attention limitations of MRDDANet, SAR-CAM employs multiple attention blocks to enable global attention across the image. They used both MSE and TV loss and trained on synthetically speckled images from the UC Merced Land Use dataset [17].

Since their introduction by Goodfellow et al. [22] in 2014, generative adversarial networks (GANs) have become a popular method of generating images and have been used extensively for despeckling SAR images. ID-GAN, proposed by Wang et al. [23], published in 2017, was the first adaptation of GAN for SAR despeckling. More recently, a new technique based on Denoising Diffusion Probabilistic Models has been able to produce more realistic images while avoiding GAN’s mode collapse [2]. When Dhawal and Nichol [3] showed that diffusion can generate images better than GANs, research into diffusion expanded rapidly and has become the backbone for the prompt-based image generation techniques seen today.

### Diffusion Models

Diffusion models work by progressively adding Gaussian noise to a clean image, then training a deep learning model to iteratively remove that noise. More specifically, the DDPM framework, as defined by [2], utilizes a Markov chain to diffuse a clean image $x_{0}$ into white Gaussian noise $x_{T}∼N\left(0,1\right)$ over *T* steps in a forward diffusion process. Over each step *t*, zero-mean Gaussian noise ϵ is added to $x_{t-1}$ according to a variance schedule set by the diffusion rate $\beta_{t}$. The distribution *q* of the forward diffusion process is

(3) $$q\left(x_{t}|x_{t-1}\right):=N\left(x_{t};\sqrt{1-\beta_{t}}x_{t-1},\beta_{t}I\right)$$

to produce *T* gradually noisier images. Instead of iteratively adding noise to $x_{0}$ in the forward diffusion process, the diffusion can be performed in a single step by expressing $q(x_{t}|x_{0})$ as a Gaussian distribution by setting $\alpha_{t}:=1-\beta_{t}$ and ${\bar{\alpha }}_{t}:=\prod_{s=0}^{t}\alpha_{s}$ [2].

(4) $$\begin{array}{cc} q(x_{t}|x_{0}) & =N(x_{t};\sqrt{\bar{\alpha_{t}}}x_{0},\left(1-\bar{\alpha_{t}}\right)I) \end{array}$$

(5) $$\begin{array}{cc} \; & =\sqrt{\bar{\alpha_{t}}}x_{0}+\epsilon \sqrt{1-\bar{\alpha_{t}}} \end{array}$$

We derive the distribution *p* of the reverse diffusion process to be

(6) $$p_{\theta }\left(x_{t-1}|x_{t}\right):=N\left(x_{t-1};\mu_{\theta }\left(x_{t},t\right),\sum_{\theta }\left(x_{t},t\right)\right)$$

The reverse process can be represented by a neural network trained to learn the mean $\mu_{\theta }$ and diagonal covariance matrix $\sum_{\theta }$ of the Gaussian distribution produced by $q(x_{t}|x_{t-1})$ at timestep *t*. The training objective for such a neural network can be defined as optimizing the variational lower bound $L_{vlb}$ for $p_{\theta }\left(x_{0}\right)$ [2]:

(7) $$L_{vlb}:=\sum_{t=0}^{T}L_{t}$$

$L_{t}$ is defined by $L_{0}$, $L_{t-1}$, and $L_{T}$:

(8) $$\begin{array}{cc} L_{0} & =-\log(p_{\theta }\left(x_{0}|x_{1}\right)) \end{array}$$

(9) $$\begin{array}{cc} L_{t-1} & =D_{KL}(q\left(x_{t-1}|x_{t},x_{0}\right)\left|\right|p_{\theta }\left(x_{t-1}|x_{t}\right)) \end{array}$$

(10) $$\begin{array}{cc} L_{T} & =D_{KL}(q\left(x_{T}|x_{0}\right)||p\left(x_{T}\right)) \end{array}$$

where $D_{KL}\left(P||Q\right)$ represents the Kullback–Leibler divergence [2].

In practice, Ho et al. [2] found that simplifying the training objective to predict the noise ϵ rather than the mean $\mu_{\theta }$ produces better results:

(11) $$L_{simple}:=E_{t,x_{0},\epsilon }\left[||\epsilon -\epsilon_{\theta }\left(x_{t},t\right){\left|\right|}^{2}\right]$$

where the predicted mean $\mu_{\theta }$ can be derived from the predicted noise $\epsilon_{\theta }$:

(12) $$\mu_{\theta }\left(x_{t},t\right)=\frac{1}{\sqrt{\alpha_{t}}}\left(x_{t}-\frac{1-\alpha_{t}}{\sqrt{1-\bar{\alpha_{t}}}}\epsilon_{\theta }\left(x_{t},t\right)\right)$$

Notably, [2] found that the covariance matrix does not need to be learned and instead, the constant upper or lower bounds, $\beta_{t}I$ and $\tilde{\beta_{t}}I$, respectively, are used for the true reverse step variance.

To generate a high-resolution image using this method, Dhariwal and Nichol [3] start with an image made entirely of Gaussian noise, denoted as $x_{T}$, and gradually refine it over *T* iterations. In each step, they pass $x_{t}$ (the noisy image at step *t*) through a modified U-Net model [15], which outputs a slightly denoised version, $x_{t-1}$. This U-Net model is conditioned on the timestep *t*, helping it estimate how much of $x_{t}$ is noise versus true signal.

For super-resolution, a low-resolution image is upscaled with bicubic interpolation, treating any interpolation errors as noise. Then, the same diffusion process is applied to refine this upscaled image, using the U-Net to progressively reduce noise. By conditioning the U-Net on both the upscaled image $x_{S}$ and a partially denoised version of the image $x_{t}$ at each step, the model gradually removes noise until it generates a clean, high-resolution image $x_{0}$.

**Figure 1:**
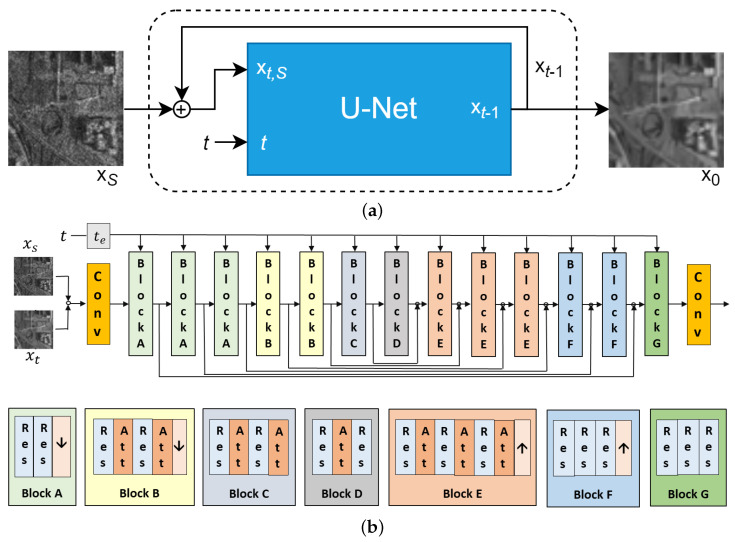
(**a**) DDPM architecture used by [4] for SAR despeckling. (**b**) Modified U-Net architecture: Conv is convolutional block; Res is ResNet block; Att is attention block; $t_{e}$ is timestep embedding; down arrow is downsampling; up arrow is upsampling.

Perera et al. proposed SAR-DDPM [4] in 2023, being the first to adapt DDPM to SAR despeckling. For the reverse diffusion process, illustrated in Figure 1, the approach in [4] used a modified U-Net architecture designed by [3] for super-resolution that was pretrained on ImageNet. These modifications to the original U-Net [15] are designed to incorporate information from a given noisy image and allow the model to better preserve important features while despeckling, instead of solely relying on learned priors. Our framework builds on these ideas by refining how intermediate denoised samples are utilized, further optimizing the balance between noise removal and detail preservation.

Here, $x_{t,S}$ represents the partially denoised image at timestep *t* concatenated with $x_{S}$. The modified U-Net shown in Figure 1b contains an encoder and decoder using repeating layers of convolution, residual blocks, and global attention blocks, along with skip connections, with further details described in [3,4]. The U-Net is conditioned on the speckled image $x_{S}$ and the partially denoised image $x_{t}$. Training used synthetically speckled images from the DSIFN dataset [24]. To enhance performance, a technique called cycle spinning is employed. This involves shifting and wrapping the image to create several modified versions. The model’s performance is evaluated on these shifted images and the results are averaged to produce a cleaner image at the expense of increased evaluation time.

Many despeckling methods, including SAR-DDPM, are limited to processing images at specific resolutions. Consequently, when applied to larger images, these methods often divide the image into smaller blocks for processing. However, this approach can introduce edge artifacts at the boundaries between blocks. To address this issue, Hu et al. [25] proposed a solution where the image is divided into overlapping patches. A DDPM is then trained on synthetically speckled images to despeckle each patch individually. By averaging the overlapping regions of neighboring patches, a smoother result was achieved with fewer artifacts.

Neither of the studies that adapt DDPM for SAR despeckling consider the time required to process the images. Due to DDPM’s iterative approach, these methods can take hundreds or even thousands of times longer to run than traditional despeckling methods. Reducing this processing time is still an open and crucial area of research to make DDPM more practical and competitive for SAR despeckling. Additionally, neither study attempts to make modifications to the DDPM that specifically enhance its ability to despeckle SAR images.

This paper proposes and compares two new methodologies that use DDPM to despeckle SAR images. One is an enhancement that is applied to the model during inference time. Another is the use of a second U-Net that is trained on all or some of the partially despeckled images produced during the reverse diffusion process.

## 3. Methodology

The DDPM denoising pipeline is often performed over 1000 iterations. However, an evaluation of 1000 steps can take more than a minute per image. Thus, we consider taking these 1000 steps in increments of 10, meaning that only 100 evaluations are made; this is a standard practice in DDPM denoising. These models were pretrained on the ImageNet dataset by Dhariwal and Nichol [3] and use 3 channels; however, for our application, these channels are duplicates of the single speckled channel being despeckled.

### 3.1. Non-Uniform Sweeps

An often-overlooked feature of DDPMs is the concept of non-uniform sweeps, introduced by [3] as a way to reduce the number of denoising steps without compromising performance. Non-uniform sweeps define the step sizes for denoising as Gaussian noise is progressively removed. Unlike the default approach, where all steps are of equal size, non-uniform sweeps allow larger steps during the initial stages (when noise removal is relatively straightforward) and smaller steps toward the latter stages (when more precision is required to remove residual noise).

For example, the default SAR-DDPM sweep involves 100 uniform steps. In contrast, a non-uniform sweep might split the process into sections with different step sizes. A split of [10, 20, 30] divides the denoising process into three sections: the first third is completed in 10 steps, the second third in 20 steps, and the final third in 30 steps, enabling finer precision at the later stages. This results in a total of 60 steps but with an emphasis on accuracy as the image approaches a nearly denoised state.

Non-uniform sweeps can significantly impact both performance and evaluation time. Larger steps early on reduce the number of overall iterations, speeding up the process. However, many small steps at the end may increase the total iterations while potentially enhancing the final denoising accuracy. It is important to note that while the model can be trained on a standard sweep, it is much more common to evaluate a model using a non-uniform sweep that provides better results.

Our work further reveals that there are benefits to early stopping and we have adjusted our sweeps accordingly. Table 1 summarizes the sweeps that were evaluated on the uniformly trained DDPM in order to identify the configuration that offers the best performance for SAR despeckling. Some sweeps contain 0’s in some sections, meaning no effort is going to be spent removing noise during those stages.

The sweeps are evaluated using a DDPM model trained on the synthetically speckled DSIFN dataset [24], configured with 100 steps and a step size of 10, resulting in a total of 1000 denoising steps. Their performance is assessed based on despeckling results for two synthetically speckled datasets. The sweep with the best performance is further compared to state-of-the-art methods and our other denoising approach based on sample aggregation presented next. Our comparisons consider both synthetically speckled despeckling performance and real SAR image despeckling performance.

### 3.2. DDPM Sample Aggregation

**Figure 2:**
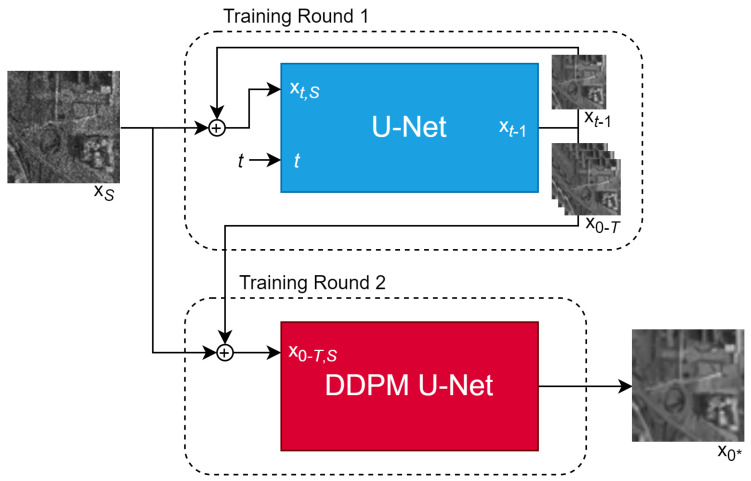
DDPM pipeline using a secondary aggregating U-Net that aggregates all partially denoised samples and the speckled image. The DDPM is trained first and then frozen during training of the aggregating U-Net.

In this section, we propose an alternative approach that aggregates all partially denoised samples generated by a trained DDPM into a secondary U-Net. The architecture for this new model is shown in Figure 2.

Here, $x_{0-T}$ defines the set of images produced by all iterations of the DDPM process, $x_{0-T,S}$ is the set of all images concatenated with the speckled image $x_{S}$, and $x_{0*}$ is the pseudo-clean image produced by the aggregating U-Net. The secondary aggregating U-Net is trained to extract a clean image from the partially denoised samples generated by the diffusion model and the original speckled image. Since some iterations may be closer to the ground truth than others, the goal of the aggregating U-Net is to identify and prioritize these more accurate samples. Additionally, it can leverage long-term patterns in the denoising process that the original DDPM architecture is unable to capture. The denoising U-Net in the original DDPM is fed a timestep *t* to understand how much noise the model should expect to remove. Since this is not needed for the aggregating U-Net, a constant value of 1.0 is fed in place of *t*.

The sample aggregation method involves two training rounds. During the first round, the DDPM is trained on a select dataset for 8000 training steps, as it would normally be trained. During the second round, the DDPM model is frozen and the aggregating U-Net is trained to produce a clean image for 5000 iterations. Both training rounds use the same dataset.

### 3.3. Training and Testing Methodology

All models under evaluation are trained on two different datasets: DSFIN [24] and Sentinel-2 [18]. Since clean SAR images are not available, these optical image datasets are used to create synthetically speckled images. While training on synthetically speckled optical images is not ideal, the lack of clean, noise-free SAR images necessitates this approach. Although SAR and optical images have significant differences in sensor characteristics, the speckle noise follows statistical properties that can be learned effectively in a synthetic setting. During dataset construction, a mathematical model of speckle noise is used to simulate real SAR speckle, reducing the domain shift between synthetic and real SAR images. This technique is widely used in SAR despeckling research, as it provides a controlled environment where ground truth is available and noise reduction can be accurately assessed.

During training, each image is converted to grayscale, randomly rotated, flipped, cropped to 256 × 256 pixels, and then artificially speckled to mimic the noise typical of single-look SAR images. The models were developed using PyTorch 3.8 14 October 2019 on a Redhat-based workstation and were trained for 8000 iterations with a batch size of 8 and a learning rate of 1 ×$10^{-4}$ using an RTX 3090 GPU.

Since the pretrained models are designed for color images, the grayscale speckled images are fed in three channels for input to the model. A validation set of 40 images was evaluated every 100 iterations, and the model with the highest Peak Signal-to-Noise Ratio (PSNR) on the validation set was saved. The performance results of SAR-DDPM are analyzed in Section 4.1. Based on those results, a modified validation methodology is used for the two methods explored in this project, which is explained in Section 4.2.

For the validation and testing datasets, each image is converted to grayscale, center-cropped to 256 × 256 pixels, and speckled with noise typical of a single-look SAR image. The despeckling performance of the proposed models, along with state-of-the-art methods for comparison, is assessed on 96 synthetically speckled images from the DSIFN dataset [24] and 200 from the Sentinel-1 dataset [18]. Additionally, the models are tested on 20 real SAR images from the Sentinel-1 dataset [18] and 20 from the High Resolution SAR Images Dataset (HRSID) [26], where both use the backscatter coefficients of the given scenes.

The proposed models are compared with other state-of-the-art SAR despeckling algorithms, including the U-Net architecture from Dhariwal and Nichol [3], MRDDANet [20], SAR-CAM [21], MONet [19], and FANS [13]. Additionally, the U-Net architecture used in DDPM is trained to predict the speckle noise of a SAR image in a single pass without any of the diffusion process. A comparison with this model evaluates the importance of the iterative aspect of diffusion and the gains made using this approach. All architectures are compared in terms of performance and evaluation time.

### 3.4. Evaluation Metrics

Synthetic despeckling performance is measured using PSNR and Structural Similarity Index Measure (SSIM)—both widely accepted metrics for evaluating denoising models when ground-truth images are available [1]. Bahara and Sahoo define these two metrics as follows:

- **Peak Signal-to-Noise Ratio**: PSNR is the logarithmic ratio of peak signal power to the noise power:
  (13) $$\mathrm{PSNR}=10\log_{10}\frac{Peak^{2}}{\mathrm{MSE}(f,\hat{f})}$$
  where $Peak_{f}$ is the maximum value of the clean image *f*, in this case 1, and MSE is defined as the sum of the squared difference of pixel values between the clean image *f* and the noisy image $\hat{f}$:
  (14) $$\mathrm{MSE}\left(f,\hat{f}\right)=\frac{1}{N}\sum_{i=0}^{N-1}{\left(f_{i}-\hat{f_{i}}\right)}^{2}$$
  A higher PSNR means better noise reduction.
- **Structured Similarity Index Measure**: SSIM evaluates the perceived similarity of two images by comparing three structural aspects: luminance *l*, chrominance *c*, and structure *s*. The SSIM between two images *x* and *y* is computed as
  (15) $$\mathrm{SSIM}\left(f,\hat{f}\right)={\left[l\left(f,\hat{f}\right)\right]}^{\alpha }\cdot {\left[c\left(f,\hat{f}\right)\right]}^{\beta }\cdot {\left[s\left(f,\hat{f}\right)\right]}^{\gamma }$$
  where α, β, and γ are parameters controlling the relative importance of luminance, contrast, and structure, typically set to 1. This can be simplified into the form
  (16) $$\mathrm{SSIM}\left(f,\hat{f}\right)=\frac{\left(2\mu_{f}\mu_{\hat{f}}+C_{1}\right)\left(2\sigma_{f\hat{f}}+C_{2}\right)}{\left(\mu_{f}^{2}+\mu_{\hat{f}}^{2}+C_{1}\right)\left(\sigma_{f}^{2}+\sigma_{\hat{f}}^{2}+C_{2}\right)}$$
  where
  − $\mu_{f}$ and $\mu_{\hat{f}}$ are the mean intensities of images *f* and $\hat{f}$.
  − $\sigma_{f}^{2}$ and $\sigma_{\hat{f}}^{2}$ are the variances of images *f* and $\hat{f}$.
  − $\sigma_{f\hat{f}}$ is the covariance between *f* and $\hat{f}$.
  − $C_{1}$ and $C_{2}$ are small constants to stabilize the division.

Since ground-truth images are not available for real SAR images, their evaluation will use the Equivalent Number of Looks (ENL) and the Edge Preservation Degree based on the Ratio of Averages (EPD-ROA). These metrics are commonly used in the field to assess how well an algorithm removes noise while preserving important image features [1]. Bahara and Sahoo define these two metrics as follows:

- **Equivalent Number of Looks**: ENL is the squared ratio of the mean $\mu_{h}$ and the standard deviation $\sigma_{h}$ of a homogeneous region *h*:
  (17) $$\mathrm{ENL}={\left(\frac{\mu_{h}}{\sigma_{h}}\right)}^{2}$$
  A higher ENL means better noise reduction within smooth regions.
- **Edge Preservation Degree based on the Ratio of Averages**: EPD-ROA was introduced by Feng et al. [27] and measures the degree to which edges are preserved when despeckling noisy image *g* into despeckled image $\hat{f}$ within a small region:
  (18) $$\mathrm{EPD}-\mathrm{ROA}=\frac{\sum_{i=1}^{N}\left|{\hat{f}}_{i,1}/{\hat{f}}_{i,2}\right|}{\sum_{i=1}^{N}\left|g_{i,1}/g_{i,2}\right|}$$
  Values closer to 1 indicate better edge preservation. The metric measures edge preservation in a particular direction; this paper opts to evaluate performance in both the vertical and horizontal directions and then average them together into a single metric.

The homogeneous and heterogeneous regions were hand-selected for each of the 20 images in the real SAR datasets. Diffusion techniques start with pure Gaussian noise, which can lead to variability when evaluating the same image multiple times. To improve the reliability of non-ground-truth metrics, such as ENL and EPD-ROA, each image in the SAR testing datasets is evaluated three times, each with a different initial Gaussian noise. The ENL and EPD-ROA values are calculated for each iteration and averaged for each image. The dataset’s overall performance is then determined by calculating the mean of these averages across all images.

To assess the consistency of the results, the coefficient of variation (CV) is used to measure variability in the ENL and EPD-ROA values across the three iterations for each image. The coefficient of variation is defined as

(19) $$CV=\frac{\sigma }{\mu }$$

where σ is the standard deviation of the metric, and μ is the mean of the metric. A lower CV value is better. The CV is particularly appropriate because ENL values can vary significantly in scale from one image to another. By normalizing the variability relative to the mean, the CV provides a standardized measure of consistency. The ENL CV and EPD-ROA CV are averaged across the entire dataset to evaluate the overall reliability of the despeckling performance.

## 4. Results and Analysis

### 4.1. Preliminary DDPM Results

Initial experimentation found that diffusion models do not seem to provide the best performance for despeckling SAR images. Table 2 shows the results of a DDPM trained to remove speckle from the synthetically speckled DSIFN training set when evaluated on the DSIFN testing set.

The table surprisingly shows that SAR-DDPM performs significantly worse than nearly all other state-of-the-art models, except for FANS which is the only non-machine learning approach. Similar results were found when this model was tested on the Sen-2 testing set and when the model trained on the Sen-2 training set was tested on DSIFN and Sen-2. Those results can be found in Table A5 and Table A6 in Appendix A, respectively.

**Figure 3:**
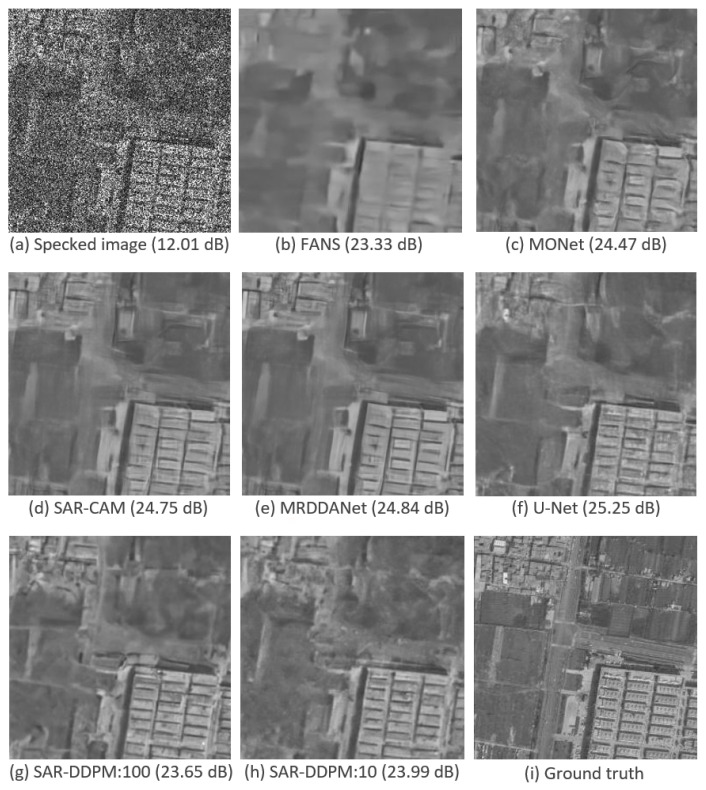
Predicted images using a sample from the DSIFN dataset with their PSNR measurements.

Figure 3 shows a side-by-side comparison of an image taken from the test set of DSIFN, evaluated by each model shown in Table 2. Interestingly, the two SAR-DDPM predictions perceptually look like they recreate the structure and texture better than the predictions from FANS, MONet, SAR-CAM, and MRDDANet. The prediction from U-Net is also very smooth, but does not have the cleaner edges found in the SAR-DDPM prediction. The two SAR-DDPM predictions are still far from perfect.

**Figure 4:**
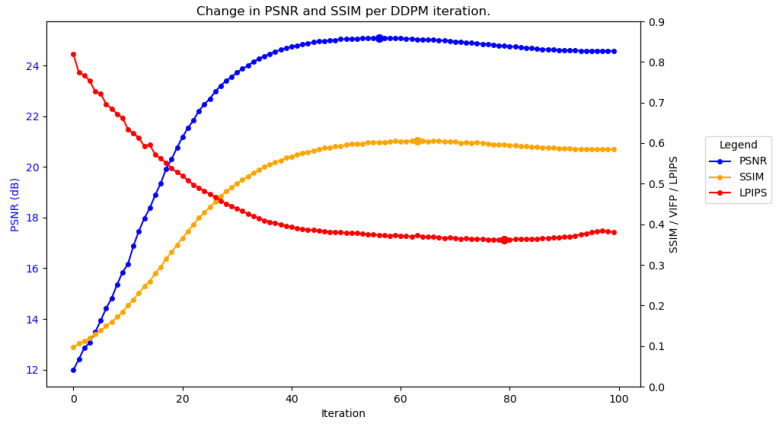
Performance over time for SAR-DDPM trained on DSIFN, tested on DSIFN, using 100 steps.

To further understand why this is the case, the accuracy of the predicted images can be measured as they become progressively cleaner at each DDPM iteration. Figure 4 plots the average performance across each iteration of SAR-DDPM trained on DSIFN, tested on DSIFN, when using 100 steps. The blue line represents the PSNR and the yellow line represents SSIM, where for both, higher is better. In the figure, both PSNR and SSIM are at their highest values about halfway through the denoising process, and begin to fall off afterwards. The maximum PSNR and SSIM are indicated by the large plot points. The red line represents LPIPS [28], which is a perceptual quality metric where lower is better. Results are not compared using LPIPS in this research since a complete perceptual analysis is out of the scope of this project, but it is included to supplement the point that PSNR and SSIM do not capture the quality of the image.

**Figure 5:**
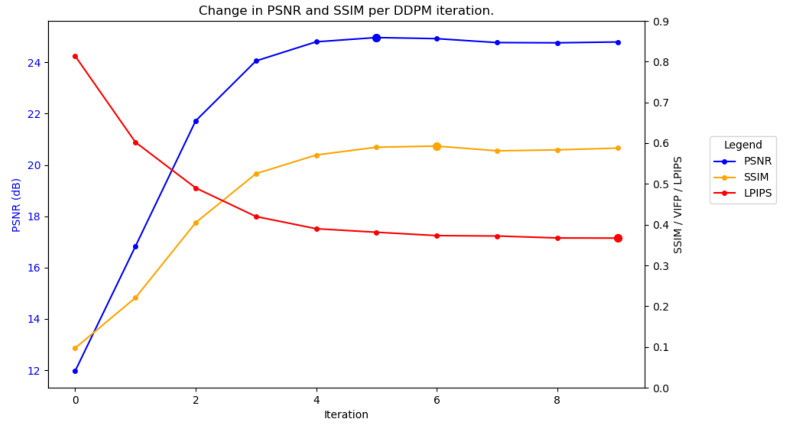
Performance over time for SAR-DDPM trained on DSIFN, tested on DSIFN, using 10 steps.

Figure 5 plots the average performance across each iteration for SAR-DDPM trained on DSIFN, tested on DSIFN, when using 100 steps. Both configurations produce a similar performance curve across their evaluation processes, showing that this performance degradation is independent of the number of steps used.

**Figure 6:**
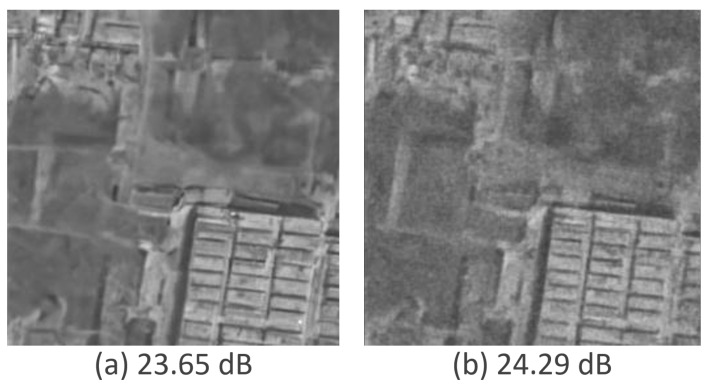
Predicted images at different iterations in the denoising process. (**a**) Sample at iteration 100. (**b**) Sample at PSNR-Max.

Figure 6 presents a sample from the DSIFN testing set generated at the final iteration of the DDPM pipeline using 100 steps, alongside the sample with the highest PSNR score within the DDPM pipeline, referenced here as the sample at PSNR-Max. The image sample at iteration 100 appears visually cleaner than the sample at PSNR-Max but has a significantly lower PSNR score. The sample at PSNR-Max still contains a noticeable amount of Gaussian noise. This suggests that diffusion models may not be ideally suited for SAR despeckling. The final iteration of the DDPM generates an image that is less accurate at the pixel level, but looks more realistic and visually appealing than the one at PSNR-Max—and arguably more realistic than all other samples shown in Figure 4.

An important observation of this paper is that DDPM excels at producing visually realistic images within a target domain, but after iteration PSNR-Max, it likely begins “hallucinating” structures and features to enhance the image’s realism as a despeckled SAR image. However, the primary goal of SAR despeckling is not to create visually realistic images, but to achieve pixel-level accuracy, consistent with the original noise-free image.

The reason generative models produce hallucinations remains an open question in AI research and is beyond the scope of this paper. We conjecture that a possible reason for hallucinations is the use of the Kullback–Leibler (KL) divergence in the loss function. The KL divergence measures how one probability distribution diverges from a second, expected probability distribution, which can lead to the generation of plausible but incorrect details in an attempt to match the learned distribution of clean images. In the case of SAR despeckling, this issue is particularly pronounced because diffusion models prioritize generating images that resemble clean SAR data rather than strictly adhering to the original noisy input. This tendency to reconstruct details based on learned priors, rather than strictly removing noise, can cause structural artifacts or unrealistic features, negatively impacting quantitative performance despite producing perceptually cleaner images.

### 4.2. Alternative Validation Results

To address this limitation, modifications can be introduced to improve the quality of the denoised output. Specifically, an alternative training approach can be implemented with a specialized validation methodology. Instead of saving the model with the best final-iteration PSNR score, the model with the highest PSNR at PSNR-Max is saved during training (evaluated at every 100 iterations). During evaluation, the iteration with the highest PSNR score is saved as the result. This adjustment significantly enhances the performance of SAR-DDPM, as shown in the experimental results summarized in Table 3 and labeled as ’SAR-DDPM-Max’.

With this method, the performance of the SAR-DDPM increases significantly to become the second-best-performing model behind U-Net. Similar results were found when testing this model on the Sen-2 testing set and when training the model on the Sen-2 training set. Those results can be found in Table A1 and Table A2 in Appendix A, respectively.

**Figure 7:**
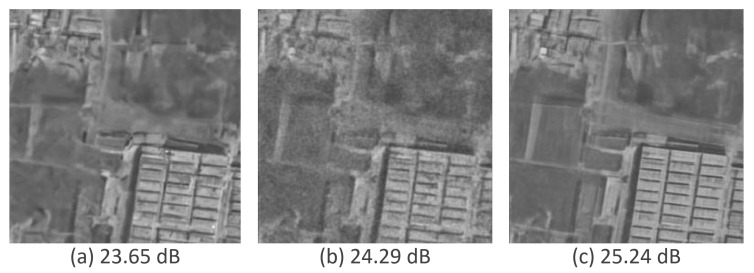
Predicted images using different validation and evaluation techniques. (**a**) Sample at iteration 100. (**b**) Sample at PSNR-Max. (**c**) Sample at PSNR-Max using specialized validation.

Figure 7 shows three samples from DSIFN using SAR-DDPM in 100 steps: one that is the last sample using the original validation methodology, one that is the sample at PSNR-Max using the original validation methodology, and one that is the sample at PSNR-Max using the specialized validation methodology.

The new validation technique clearly produces images that are much smoother with cleaner edges than those produced from the original validation technique. However, simply extracting the best sample from the DDPM pipeline is not a scalable solution for despeckling real SAR images, as these images lack a ground truth for comparison, and decisions would rely solely on subjective judgments of visual quality. To address this challenge, this research explores systematic methods for identifying and extracting the best sample from all iterations of the DDPM pipeline. These methods include strategies such as early stopping during evaluation using sweeps, and training an additional model specifically designed to identify and extract the optimal sample from the iterations generated by DDPM.

### 4.3. DDPM Sweep Results

To determine the best method for evaluating speckled images using DDPM, a variety of uniform and non-uniform sweeps were tested and compared. They were all evaluated on the same model trained on the DSIFN dataset and using the specialized validation process to produce the cleanest possible samples. The results are shown in Table 4.

Of the uniform sweeps, a sweep of 5 steps performs the best in both PSNR and SSIM, but this is likely due to the fact that fewer steps means that the model has fewer opportunities to hallucinate features that are not there. Of the non-uniform sweeps, the sweep of [8,8,12,0,0,0] performs the highest in both PSNR and SSIM, and uses only half of the sampling steps as [16,16,24,0,0,0], while also greatly outperforming all of the uniform sweeps. The same experiment was performed on the DSIFN testing set, the results from which are shown in Table A3 in Appendix A. Similar results were found when the models were trained on the Sentinel-2 training set, which can be found in Table A4 in Appendix A.

**Figure 8:**
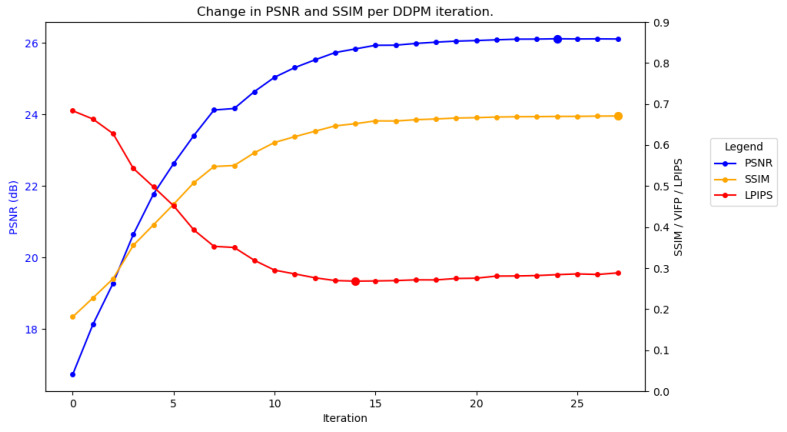
Performance over time for SAR-DDPM trained on DSIFN, tested on DSIFN, using the best sweep.

Figure 8 plots the performance of SAR-DDPM over time when evaluating images using a sweep of [8,8,12,0,0,0]. The plot shows that this method provides an adequate number of steps to denoise the Gaussian noise into a very accurate result, while preventing the DDPM from hallucinating features. Additionally, by averaging the last eight samples in the sweep [8,8,12,0,0,0], we are able to further improve performance. Those results are summarized in Table 5.

**Figure 9:**
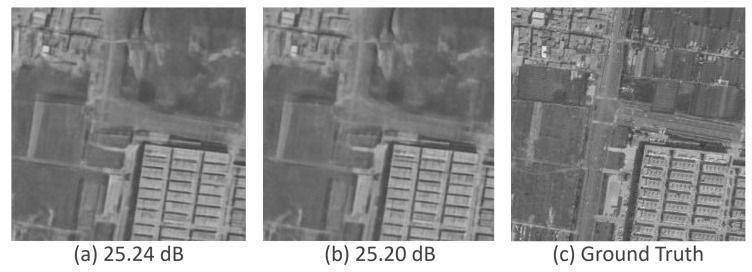
Predicted images using max sample versus the best sweep method. (**a**) Sample at PSNR-Max using specialized validation. (**b**) Sample using best sweep. (**c**) Ground truth.

Figure 9 shows the sample at PSNR-Max using the specialized validation methodology, the sample produced by averaging the last 8 samples from the sweep of [8,8,12,0,0,0], and the ground truth.

The two samples are nearly identical in appearance and have very similar PSNR scores. Notably, the iteration at which PSNR-Max occurs may vary between images. However, using a standardized sweep for evaluation—applied consistently across all images—achieves performance comparable to selecting the best sample from all iterations. This demonstrates the effectiveness of the sweep in adapting the DDPM for SAR despeckling, making it a reliable and practical modification.

The metric results for this method, compared to the state-of-the-art results, are shown in Table 6. This method is a state-of-the-art diffusion technique for despeckling SAR images, but still falls short of the performance of U-Net denoising.

### 4.4. DDPM Sample Aggregation Results

Another approach that we tested involves training a secondary U-Net to aggregate and refine the output sample from the set of DDPM iterations. This method aims not only to select the best iteration but also to improve upon the despeckling results. To train this second U-Net, images were evaluated on the original DDPM using two configurations: 100 iterations and 10 iterations. The results of these aggregation evaluations are summarized in Table 7. The results for the models evaluated using 100 iterations and 10 iterations are labeled ’SAR-DDPM Agg.:100’ and ’SAR-DDPM Agg.:10’, respectively.

The performance of this second U-Net exceeds that of the standalone DDPM, both with and without sweeps, making it another state-of-the-art solution to despeckling SAR images. Both configurations, the one utilizing 100 iterations and the other utilizing 10, perform very similarly, indicating that using more iterations is not necessarily beneficial. However, this model does not outperform the standalone U-Net, meaning that the iterative approach of diffusion models may hinder the capabilities of the U-Net even with the two improvements introduced in this paper. Similar results were found when evaluating on the Sen-2 test set, and when the models were trained on the Sentinel-2 training set, which can be found in Table A5 and Table A6 in Appendix A, respectively.

**Figure 10:**
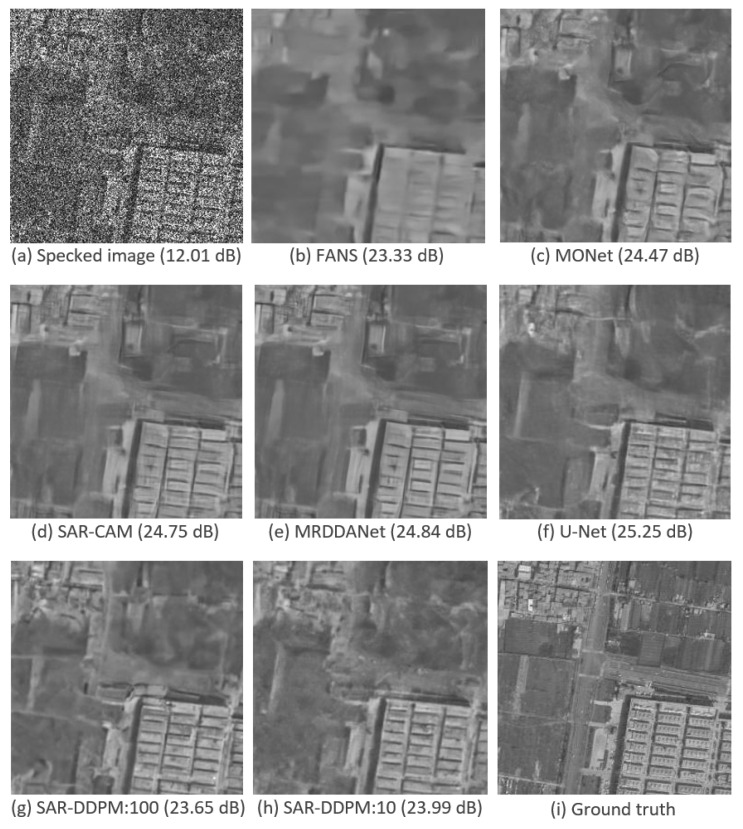
Predicted images using a sample from the DSIFN dataset with their PSNR measurements. DDPM Agt. uses 100 iterations.

Figure 10 shows a side-by-side comparison of an image taken from the test set of DSIFN, evaluated by each model shown in Table 7. While the PSNR and SSIM scores for the sweep and aggregation methods may be lower than those of U-Net, the images produced by the sweep and aggregation methods are still perceptually cleaner than the ones produced by every state-of-the-art model, including U-Net.

**Figure 11:**
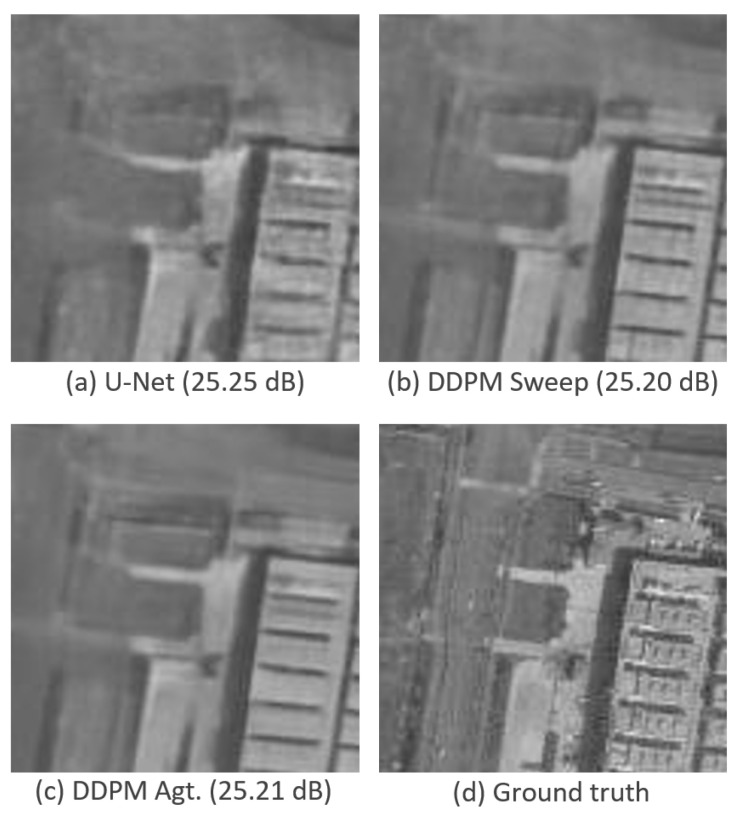
Close-up of predictions using a sample from the DSIFN dataset with their PSNR measurements. DDPM Agt. uses 100 iterations.

Figure 11 shows a close-up of the image in Figure 10 for U-Net, SAR-DDPM Sweep, and SAR-DDPM Agg.:100 to make comparisons easier. The two SAR-DDPM methods both produce a cleaner image than the one produced by the standalone U-Net. The edges are clearly sharper and straighter, and the regions are smoother and less noisy. Notably, the SAR-DDPM Sweep image attempts to preserve some of the additional texture seen on top of the buildings in the bottom right, while the SAR-DDPM Aggregation image smooths that region very well.

The close-up images in Figure 11 highlight that our proposed DDPM-based methods produce cleaner edges and smoother textures compared to U-Net, even though the quantitative metrics favor U-Net. One can see in Figure 11 that the denoised image produced by U-Net has edges that are not straight and homogeneous areas that are not smooth compared to the denoised images produced by our proposed models. This discrepancy between perceptual quality and standard despeckling metrics is a well-known challenge in image denoising and is an area of future research. Traditional metrics such as PSNR and SSIM do not fully capture perceptual quality in SAR despeckling where structural integrity and noise suppression must be balanced.

### 4.5. Real SAR Despeckling Results

Finally, all methods were evaluated on real SAR images: 20 from Sentinel-1 and 20 from HRSID. The results of the SAR tests that were produced by models trained on DSIFN are shown in Table 8. Note that the CV metrics only apply to the SAR-DDPM models because they start with pure Gaussian noise, which can change the predicted image drastically. CV measures the ability for a model to produce consistent results, and a lower value is desired.

All SAR-DDPM configurations performed exceptionally well on the Sentinel-1 test. The aggregation approach excelled in the ENL metric, achieving state-of-the-art or near-state-of-the-art results, while the vanilla approach demonstrated similarly strong performance in the EPD-ROA metric. As for HRSID, the SAR-DDPM approaches all achieved good but not great ENL scores (except for SAR-DDPM:100 which surprisingly scored exceptionally well). For EPD-ROA, all models performed well, especially for the sweep and aggregate approaches, which achieved nearly state-of-the-art performance.

The ENL CV scores for both SAR-DDPM Agg. configurations were lower than that of SAR-DDPM Sweep, and much lower than the two standard SAR-DDPM configurations, meaning that the sample aggregation approach produces very stable results. For all SAR-DDPM variations, the EPD-ROA CV scores all were below 0.010, meaning that all were very consistent for this metric. Similar results were found when the models were trained on the Sentinel-2 dataset, which can be found in Table A7 in Appendix A.

**Figure 12:**
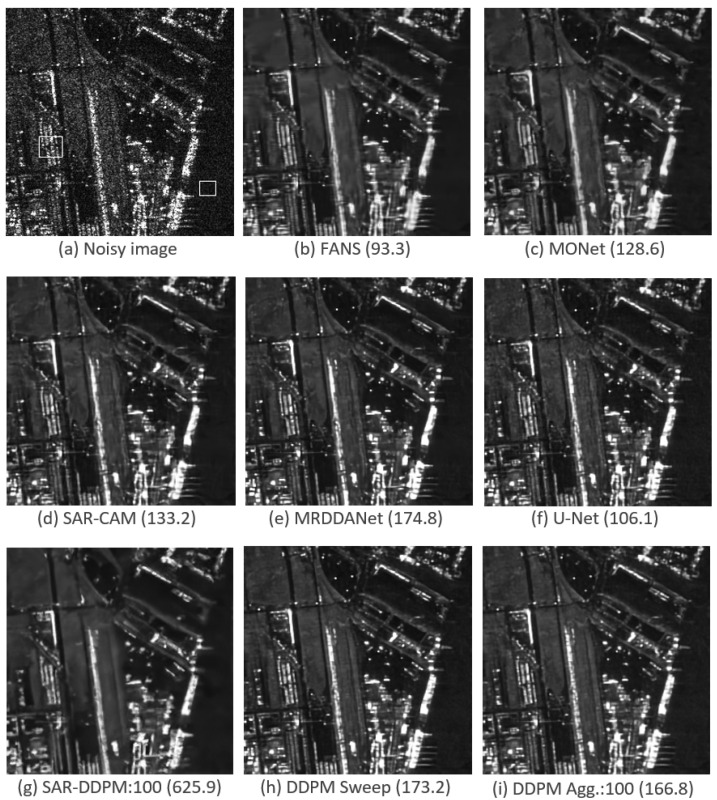
Predicted images using a sample from the HRSID dataset with their ENL scores.

Figure 12 shows a side-by-side comparison of an image taken from HRSID, evaluated by each model shown in Table 8. In the original SAR image, the left box defines the heterogeneous region used for EPD-ROA, and the right box defines the homogeneous region used for ENL.

Interestingly, the model that produced the image with the greatest ENL was the one produced by SAR-DDPM:100 without any additional modifications. SAR-DDPM Sweep and SAR-DDPM Agg.:100 performed very well, smoothing out the image in homogeneous regions but keeping structure in heterogeneous ones, but MRDDANet still produced a better result here.

**Figure 13:**
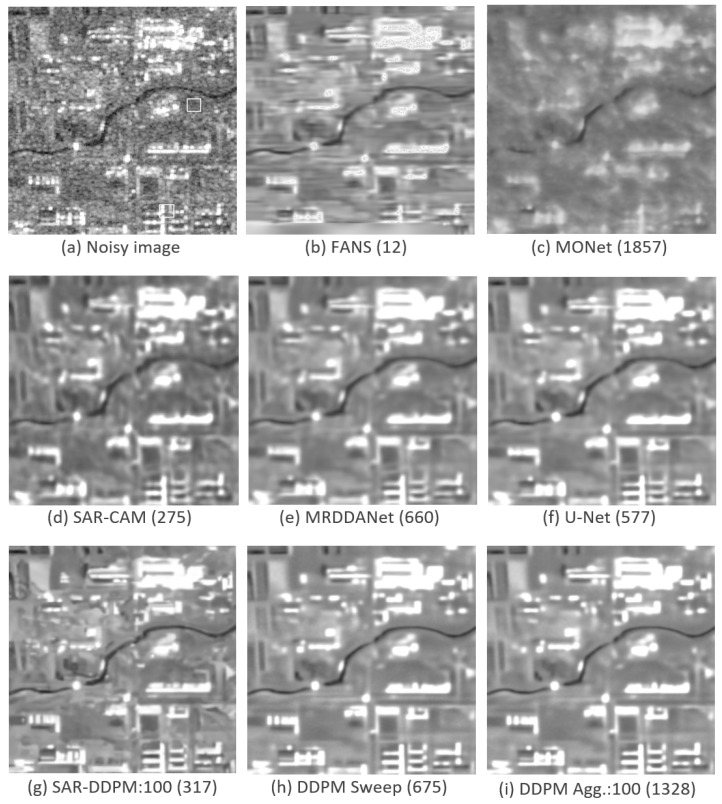
Predicted images using a sample from the Sentinel-1 dataset with their ENL scores.

Figure 13 shows the same comparison for an image taken from the Sentinel-1 dataset, evaluated by each model shown in Table 8. These images have significantly lower resolution compared to images from HRSID. In the unfiltered image, the center right box defines the homogeneous region used for ENL, and the box at the bottom defines the heterogeneous region used for EPD-ROA.

In this particular image, MONet achieves the highest ENL value, but the resulting image appears significantly oversmoothed and smeary, indicating that ENL does not always determine the best result. The images with the next highest ENL values are produced by the two proposed DDPM methods. Although the images look nearly identical, the aggregating method achieves a noticeably higher ENL value. Interestingly, the unmodified SAR-DDPM model, which produced the best result for the HRSID image shown in Figure 12, yields a relatively poor ENL value in this case. Moreover, it is evident that this model introduces hallucinated features that are absent from any other pseudo-clean image.

### 4.6. Inference Time Results

The benefits of an iterative approach to SAR despeckling over a single-pass one cannot be analyzed without also considering the amount of time it takes for each model to evaluate an image. Table 9 includes the time to evaluate a single image for each model.

MONet, U-Net, SAR-CAM, and MRDDANet all had very low evaluation times because they are single-pass CNN-based models, while FANS had a much greater evaluation time since its approach involved matching each image patch with many other image patches in the image.

For the DDPM-based approaches, the evaluation time was linearly proportional to the number of passes taken. By reducing the number of samples from 100 to 10, the evaluation time is reduced by a factor of 10, while the performance stays the same or even improves in some metrics. With this in mind, SAR-DDPM Agg. using 10 samples is the ideal model, since it performs very well in both despeckling performance and evaluation time.

## 5. Conclusions and Future Work

This research demonstrates that diffusion models, particularly SAR-DDPM and its proposed modifications, offer a highly effective approach to SAR despeckling when trained on synthetically speckled datasets. Enhancements such as non-uniform sweeps and an aggregating U-Net achieve performance on par with or exceeding state-of-the-art methods for both synthetic and real SAR imagery.

While models like MRDDANet excelled in specific real SAR metrics, SAR-DDPM variants delivered a superior balance by producing smoother textures in homogeneous regions and preserving structural details in heterogeneous areas, a strength often lacking in competing methods. Hallucinations remain a primary challenge when using DDPM for SAR despeckling, and further research is needed to investigate the underlying causes and conditions for these hallucinations.

Although diffusion models are slower than some state-of-the-art techniques, the optimizations introduced in this work reduce evaluation time without compromising image quality. Moreover, SAR-DDPM excelled in generating perceptually superior images, outperforming all other methods in visual quality. However, current metrics such as PSNR and SSIM fail to fully capture these perceptual improvements, highlighting the need for metrics that are better aligned with human visual assessment.

In conclusion, this research improved the performance of diffusion models for SAR despeckling, demonstrating their potential for both synthetic and real-world applications. While diffusion models produce images that appear cleaner and closer to the ground truth visually, their metric scores are often worse. Future work could explore metrics that better capture perceptual similarity between images (for synthetic datasets) or perceptual quality of individual images (for real SAR data). However, careful consideration is needed when incorporating these metrics, as the goal of denoising is to balance both pixel-level accuracy and the perceptual quality of the output images.

## Figures and Tables

**Table 1:** Different variations of tested DDPM sweeps.

Sweep Type	Sweep Schedule	Total Steps
Uniform	100	100
Uniform	10	10
Non-uniform: coarse to fine	10, 20, 30, 40	100
Non-uniform: fine to coarse	40, 30, 20, 10	100
Non-uniform: fine to coarse	20, 20, 10, 10	60
Non-uniform: fine to coarse	20, 20, 5, 5	50
Uniform: early stopping	20, 20, 0, 0	40
Non-uniform: early stopping	16, 16, 24, 0, 0, 0	56
Non-uniform: early stopping	8, 8, 12, 0, 0, 0	28

**Table 2:** DSIFN synthetic dataset despeckling performance for models trained on the DSIFN dataset. ’SAR-DDPM:100’ uses 100 samples during testing and ’SAR-DDPM:10’ uses 10 samples during testing **Bold** is the best score; underline is the second-best score.

	PSNR (dB)	SSIM
FANS	24.54	0.226
MONet	25.70	0.635
SAR-CAM	25.92	0.652
MRDDANet	26.08	0.665
U-Net	**26.25**	**0.676**
SAR-DDPM:100	24.59	0.584
SAR-DDPM:10	24.80	0.588

**Table 3:** Comparative DSIFN synthetic dataset despeckling performance of ’SAR-DDPM-Max’ when trained on the DSIFN dataset. **Bold** is the best score; underline is the second-best score.

	PSNR (dB)	SSIM
MONet	25.70	0.635
SAR-CAM	25.92	0.652
MRDDANet	26.08	0.665
U-Net	**26.25**	**0.676**
SAR-DDPM:100	24.59	0.584
SAR-DDPM:10	24.80	0.588
SAR-DDPM-Max:100	26.15	0.672
SAR-DDPM-Max:10	26.07	0.668

**Table 4:** Results from various DDPM sweeps when trained on DSIFN and tested on the DSIFN testing set. **Bold** is the best score; underline is the second-best score.

Sweep Schedule	Total Steps	PSNR	SSIM
100	100	24.04	0.569
10	10	25.28	0.632
5	5	25.52	0.649
[10, 20, 30, 40]	100	23.85	0.559
[40, 30, 20, 10]	100	24.50	0.595
[20, 20, 10, 10]	60	24.55	0.596
[20, 20, 5, 5]	50	24.96	0.617
[20, 20, 0, 0]	40	26.10	0.671
[16, 16, 24, 0, 0, 0]	56	**26.11**	**0.672**
[8, 8, 12, 0, 0, 0]	28	**26.11**	0.671

**Table 5:** Results from averaging samples in the sweep of [8,8,12,0,0,0] when trained on DSIFN and tested on the DSIFN testing set. **Bold** is the best score.

Sample to Evaluate	PSNR	SSIM
Last sample	26.11	0.671
Average of last 8 samples	**26.14**	**0.672**

**Table 6:** DSIFN synthetic dataset despeckling performance for models trained on the DSIFN dataset. The averaged sweep is labeled as ’SAR-DDPM Sweep’. **Bold** is the best score; underline is the second-best score.

	PSNR (dB)	SSIM
FANS	24.54	0.226
MONet	25.70	0.635
SAR-CAM	25.92	0.652
MRDDANet	26.08	0.665
U-Net	**26.25**	**0.676**
SAR-DDPM:100	24.59	0.584
SAR-DDPM:10	24.80	0.588
SAR-DDPM Sweep	26.14	0.672

**Table 7:** DSIFN synthetic dataset despeckling performance for models trained on the DSIFN dataset. **Bold** is the best score; underline is the second-best score.

	PSNR (dB)	SSIM
FANS	24.54	0.226
MONet	25.70	0.635
SAR-CAM	25.92	0.652
MRDDANet	26.08	0.665
U-Net	**26.25**	**0.676**
SAR-DDPM:100	24.59	0.584
SAR-DDPM:10	24.80	0.588
SAR-DDPM Sweep	26.14	0.672
SAR-DDPM Agg:100	26.21	0.674
SAR-DDPM Agg:10	26.22	0.675

**Table 8:** SAR despeckling performance for models trained on the DSIFN dataset. **Bold** is the best score; underline is the second-best score.

	Sentinel-1				HRSID			
	ENL	ENL CV	EPD-ROA	EPD CV	ENL	ENL CV	EPD-ROA	EPD CV
FANS	3.8		1.242		61.8		0.730	
MONet	315.6		1.333		195.2		0.726	
SAR-CAM	129.1		1.009		133.7		0.731	
MRDDANet	**403.1**		2.379		141.8		**0.740**	
U-Net	271.5		0.957		99.5		0.737	
SAR-DDPM:100	152.7	0.212	**1.000**	0.005	**253.7**	0.353	0.726	0.008
SAR-DDPM:10	177.3	0.168	0.998	**0.003**	130.1	0.294	0.723	0.006
SAR-DDPM Sweep	286.6	0.069	1.057	0.008	115.3	0.118	0.736	0.003
SAR-DDPM Agg.:100	317.6	**0.051**	1.058	0.007	138.4	0.078	0.734	**0.002**
SAR-DDPM Agg.:10	320.3	0.065	1.055	0.006	103.5	**0.053**	0.735	**0.002**

**Table 9:** Average evaluation times of each model. **Bold** is the best time; underline is the second-best time.

	Time (sec)
FANS	1.44
MONet	**0.07**
SAR-CAM	0.09
MRDDANet	0.11
U-Net	0.08
SAR-DDPM:100	7.21
SAR-DDPM:10	0.72
SAR-DDPM Sweep	2.03
SAR-DDPM Agg:100	7.30
SAR-DDPM Agg:10	0.78

## Data Availability

The code used to train and evaluate the models proposed in this project can be found online at https://github.com/asp6244/SAR-DDPM-Aggregation (accessed on 12 March 2025).

## Abbreviations

The following abbreviations are used in this manuscript:

DDPM	Denoising Diffusion Probabilistic Model
SAR	Synthetic Aperture Radar
CNN	Convolutional Neural Network
GAN	Generative Adversarial Network
PSNR	Peak Signal to Noise Ratio
SSIM	Structural Similarity Index Measure
ENL	Equivalent Number of Looks
EPD-ROA	Edge Preservation Degree based on the Ratio of Averages
CV	Coefficient of Variation
TV	Total Variation
FANS	Fast Adaptive Nonlocal SAR despeckling
MONet	Multi-Objective Network
CAM	Continuous Attention Module
MRDDANet	Multiscale Residual Dense Dual Attention Network

## Appendix A

**Table A1:** Comparative synthetic dataset despeckling performance of SAR-DDPM-Max when trained on DSIFN dataset. **Bold** is best score; underline is second-best score.

	DSIFN		Sentinel-2	
	PSNR (dB)	SSIM	PSNR (dB)	SSIM
SAR-DDPM:100	24.59	0.584	23.97	0.585
SAR-DDPM:10	24.80	0.588	24.16	0.590
SAR-DDPM-Max:100	**26.15**	**0.672**	**25.98**	**0.695**
SAR-DDPM-Max:10	26.07	0.668	25.83	0.692

**Table A2:** Comparative synthetic dataset despeckling performance of SAR-DDPM-Max when trained on Sen-2 dataset. **Bold** is best score; underline is second-best score.

	DSIFN		Sentinel-2	
	PSNR (dB)	SSIM	PSNR (dB)	SSIM
SAR-DDPM:100	24.59	0.584	23.97	0.585
SAR-DDPM:10	24.80	0.588	24.16	0.590
SAR-DDPM-Max:100	**26.00**	**0.662**	**26.07**	**0.701**
SAR-DDPM-Max:10	25.93	0.659	25.88	0.697

**Table A3:** Test results from various DDPM sweeps when trained on DSIFN. **Bold** is best score; underline is second-best score.

		DSIFN		Sentinel-2	
Sweep Schedule	Total Steps	PSNR	SSIM	PSNR	SSIM
100	100	24.04	0.569	23.78	0.592
10	10	25.28	0.632	25.05	0.657
5	5	25.52	0.649	25.06	0.668
[10, 20, 30, 40]	100	23.85	0.559	23.62	0.582
[40, 30, 20, 10]	100	24.50	0.595	24.24	0.618
[20, 20, 10, 10]	60	24.55	0.596	24.27	0.619
[20, 20, 5, 5]	50	24.96	0.617	24.72	0.641
[20, 20, 0, 0]	40	26.10	0.671	**25.89**	**0.695**
[16, 16, 24, 0, 0, 0]	56	**26.11**	**0.672**	25.88	0.694
[8, 8, 12, 0, 0, 0]	28	**26.11**	0.671	25.86	0.694

**Table A4:** Test results from various DDPM sweeps when trained on Sen-2. **Bold** is best score; underline is second-best score.

		DSIFN		Sentinel-2	
Sweep Schedule	Total Steps	PSNR	SSIM	PSNR	SSIM
100	100	23.94	0.566	23.84	0.598
10	10	25.13	0.624	25.10	0.662
5	5	25.38	0.643	24.95	0.672
[10, 20, 30, 40]	100	23.80	0.557	23.68	0.588
[40, 30, 20, 10]	100	24.37	0.590	24.32	0.624
[20, 20, 10, 10]	60	24.39	0.590	24.34	0.624
[20, 20, 5, 5]	50	24.82	0.610	24.80	0.646
[20, 20, 0, 0]	40	**25.95**	0.661	25.94	**0.699**
[16, 16, 24, 0, 0, 0]	56	**25.95**	**0.662**	**25.95**	**0.699**
[8, 8, 12, 0, 0, 0]	28	**25.95**	**0.662**	25.94	**0.699**

**Table A5:** Synthetic dataset despeckling performance for models trained on the DSIFN dataset. **Bold** is best score; underline is second-best score.

	DSIFN		Sentinel-2	
	PSNR (dB)	SSIM	PSNR (dB)	SSIM
FANS	24.54	0.226	24.89	0.395
MONet	25.70	0.635	25.97	0.699
SAR-CAM	25.92	0.652	26.22	0.714
MRDDANet	26.08	0.665	**26.31**	**0.720**
U-Net	**26.25**	**0.676**	26.14	0.704
SAR-DDPM:100	24.59	0.584	23.97	0.585
SAR-DDPM:10	24.80	0.588	24.16	0.590
SAR-DDPM Sweep	26.14	0.672	25.86	0.694
SAR-DDPM Agg.:100	26.21	0.674	25.89	0.695
SAR-DDPM Agg.:10	26.22	0.675	25.91	0.697

**Table A6:** Synthetic dataset despeckling performance for models trained on Sen-2 dataset. **Bold** is best score; underline is second-best score.

	DSIFN		Sentinel-2	
	PSNR (dB)	SSIM	PSNR (dB)	SSIM
FANS	24.54	0.226	24.89	0.395
MONet	25.64	0.633	26.10	0.709
SAR-CAM	25.54	0.624	26.15	**0.711**
MRDDANet	25.59	0.623	26.18	0.708
U-Net	26.04	0.663	**26.28**	0.710
SAR-DDPM:100	23.91	0.561	23.92	0.599
SAR-DDPM:10	25.14	0.622	25.14	0.661
SAR-DDPM Sweep	25.93	**0.699**	25.97	0.662
SAR-DDPM Agg.:100	26.06	0.666	26.06	0.704
SAR-DDPM Agg.:10	**26.10**	0.666	25.96	0.700

**Table A7:** SAR despeckling performance for models trained on the Sen-2 dataset. **Bold** is best score; underline is second-best score.

	Sentinel-1				HRSID			
	ENL	ENL CV	EPD-ROA	EPD CV	ENL	ENL CV	EPD-ROA	EPD CV
FANS	3.8		1.242		61.8		0.730	
MONet	208.9		1.282		105.1		0.729	
SAR-CAM	198.0		1.034		149.6		0.734	
MRDDANet	**367.9**		1.110		**184.2**		0.733	
U-Net	277.1		**0.968**		73.5		0.742	
SAR-DDPM:100	168.4	0.263	1.062	0.012	48.0	0.196	**0.750**	0.008
SAR-DDPM:10	240.3	0.191	1.054	0.010	86.7	0.141	0.738	0.004
SAR-DDPM Sweep	234.2	0.067	1.044	**0.005**	80.0	0.121	0.735	0.003
SAR-DDPM Agg.:100	316.5	**0.044**	1.032	0.009	75.2	0.082	0.738	0.002
SAR-DDPM Agg.:10	282.2	0.071	1.039	0.006	77.6	**0.051**	0.733	**0.001**
